# Acute Distal Biceps Tendon Repair With Cortical Button Offers Good Functional Outcomes: A Retrospective Study Focusing on Range of Motion, Muscle Strength and Pain

**DOI:** 10.7759/cureus.60343

**Published:** 2024-05-15

**Authors:** Muhammad Mannan, Usman Hafeez, Pantelis Tsantanis, Serajdin Ajnin

**Affiliations:** 1 Orthopaedic Surgery, Sheikh Zayed Medical College/Hospital, Rahim Yar Khan, PAK; 2 Trauma and Orthopaedics, University Hospital Birmingham NHS Foundation Trust, Birmingham, GBR

**Keywords:** full thickness dbt rupture, upper limb injuries, mep score, distal biceps tendon (dbt) repair, functional outcome

## Abstract

Background

Distal biceps tendon (DBT) rupture is not one of the most common upper limb injuries. Surgical intervention is recommended for these injuries to restore muscular strength and functionality. Multiple different techniques are documented in the literature, however there is no definitive consensus on the most effective surgical treatment. The objective of this study was to assess the functional results of patients who underwent repair of DBT utilizing cortical button fixation procedures.

Methods

This study is a retrospective single-unit case series consisting of 54 patients who underwent DBT repair at Heartlands Hospital in Birmingham, United Kingdom. The patients' functional outcomes was assessed by the Mayo Elbow Performance Score (MEPS).

Results

The mean age was 51±11.01 years. Patients were operated on 4.72±7.083 days after the injury. The mean pain Visual Analogue Scale (VAS) 6 months after the surgery was 0.54±0.50.

At 6 months follow-up, the average extension deficit was 2.69° (0-10), flexion 132° (120-140), supination76° (50- 85), and 77° for pronation (78-95). Patients were followed up routinely for 6 months. Mayo Elbow Performance (MEP) Score was utilized to assess the functional outcome and the mean MEP score was 91.43±8.26 which showed excellent functional outcomes for the cohort.

Conclusion

DBT repair with cortical button fixation yielded favorable functional outcomes at 6 months, notably restoring supination strength. This approach offers anatomical reinsertion while minimizing nerve damage risk.

## Introduction

The rate of DBT rupture is quite infrequent, as shown by reported rates ranging from 0.9 to 1.8 per 100,000 individuals annually. This relatively rare injury often impacts male patients of 40 to 60 years, with a documented occurrence rate of 1.2 ruptures per 100,000 individuals annually [1]. The pathological process of the injury has been ascribed to factors such as the progression of age, reduced blood supply to the tendon, and inflammation in the radial bursa, sometimes accompanied by an abrupt eccentric strain on the tendon. Nevertheless, the exact cause of the damage remains uncertain [2]. While the diagnosis of DBT rupture is often clear, there is an ongoing dispute on the appropriate surgical procedures, relevant operative anatomy, the choice between single- and double-incision surgery, and the available fixation alternatives [3].

Non-operative treatment is also possible in selective cases like old aged, low-demand patients, and surgically unfit candidates, although it can result in considerable weakness in forearm supination and elbow flexion. Surgery has generally offered increased functional outcomes [4]. Literature has documented several surgical approaches and fixation methods, including single-incision techniques and 2-incision techniques. Each method carries its own strengths and weaknesses. The selection of various approaches is primarily influenced by the surgeon's personal choice and level of comfort with the chosen strategy. The most often utilized method is the two-incision method established by the Boyd-Anderson procedure [5]. Research has shown that surgical intervention for acute ruptures of the DBT leads to enhanced elbow flexion and supination in comparison to non-operative approaches [2]. It has been observed though that a significant proportion of patients were able to attain 90% strength of the contralateral limb after the restoration of chronic distal biceps tendon ruptures [6]​​​​​​.

Obtaining a better knowledge of clinical outcomes following surgical intervention for DBT rupture is critical in determining potential treatment regimes. The main aim of this study is to assess the effectiveness of immediate repair of the distal biceps tendon (DBT) using cortical button fixation, with regards to enhancing the range of motion, muscle strength, and alleviating pain in patients. The secondary aim of this study is to evaluate the frequency of post-operative problems.

## Materials and methods

This retrospective study was carried out in Heartlands Hospital, Birmingham, UK. A total of 54 patients who underwent two‑incision repair of the DBT by a single senior surgeon in our Trauma Unit between 2017 and 2023 were included in the study. Individuals between the ages of 30 and 80 who had a radiologically verified acute DBT rupture and were eligible for surgical repair were included. The exclusion criteria encompassed individuals with a history of prior elbow surgery, chronic distal biceps tendon ruptures, and systemic illnesses that impede tendon recovery. Stratified sampling techniques were employed for the data collection.

All patients had a radiological confirmation of full-thickness DBT rupture. Radiological investigations employed for confirmation of biceps tear were Ultrasound and MRI. A portable goniometer was used to assess the passive and active range of motion of the elbow and forearm. At each follow-up, the distance from the elbow crease to the biceps muscle belly was measured, specifically during the biceps crease interval. Supination strength was assessed starting from the sixth week using a pronation-supination dynamometer while the elbow was fully supinated and flexed at a 90-degree angle. Strength data were recorded as a proportion relative to the side opposite to the dominant side. The assessment of functionality included the use of the Mayo Elbow Performance Score (MEPS). The MEPS is a commonly used metric for assessing the elbow's functionality. The scoring system is administered by clinicians and has four distinct areas, including pain, mobility, stability, and the capacity to execute five functional activities. A radiographic assessment was conducted at two weeks and three months postoperatively in order to verify the accurate placement of the button. CT scan was performed at 6 months to assess the placement of the implant and the impact of the button on the front part of the cortex, by comparing it to the radiographs taken before the surgery. In addition, the closure of the cortex surrounding the tendon and the response of the cortex to the button were assessed by measuring the circumference of the drill hole at the outer boundary of the anterior cortex and the button.

Surgical technique

No tourniquet was used in all cases, a 3-cm horizontal incision over the forearm was used for surgical exploration. It was extending from the middle of the forearm to the lateral aspect and was located 3 cm distal to the elbow crease. A second incision to deliver the retracted stump was not needed in any case, the retracted stumps would be delivered by milking the biceps muscle, this was possible in all cases because a tourniquet was not used. The bicep tendon was prepared to be healthy tissue. (A number 2 fibre loop was applied around at least 30 mm of the tendon in a whipstitch pattern coming out at the distal end of the tendon, followed by measuring the tendon diameter). A 3.2 mm drill pin was inserted into the radial tuberosity with a 45° angle to the bone while the wrist was held in hypersupination. In order to avoid iatrogenic injury to the posterior intra-osseous nerve, the drill guide was carefully placed. The drill pin was then over-reamed with the same measured tendon diameter into the anterior side cortex to enable insertion of the device. In cases where the guide wire and reamer could not be drilled into the posteromedial facet of tuberosity at a steep angle to reach the original tendon insertion location, the aim was for the site to be as close as possible. It was then used to reattach the tendon ulnarly while preserving the tuberosity cam. 500 ml of saline was used for a thorough debridement. The button was subsequently inserted into the string loop suture-tendon assembly's free suture ends using a mosquito clamp and then inserted using a button inserter. Pulling on both sutures at the same time allowed the button to be centred under the bone tunnel. The button flips in this manner to engage the radial tuberosity's anterior cortex. After the button was properly positioned, the tension slide method was used to draw each suture independently, bringing the tendon into the radius. Peek tenodesis screws were available in the set but were not needed because all tendons were ducked in a snug fit canal, Fluoroscopy was used to verify appropriate final button positioning. Further irrigation and hemostasis were carried out before the wounds were closed in layers. (Physiotherapy was initiated after two weeks with active and passive elbow movement). The process of muscle strengthening started six weeks after surgery. After six months, unrestricted lifting was permitted and patients were advised to return to contact sports six months post-op.

## Results

Results of the study demonstrated that the mean age of the patients was 51±11.01years (range 34-79 years). Demographic data is summarized in Table 1. The dominant side was the right side in the majority of the patients (92.6%) and the surgical side was also right in 48% of the patients. Most of the patients` occupation was office-based followed by manual labour workers, the majority were active in the gym. The most common mechanism of rupture was eccentric stretch while lifting (44.4%) patients.

**Table 1:** Demographic details of the patients

	Frequency	Percent
Dominant Side		
Right	50	92.6
Left	4	7.4
Surgical Side		
Right	26	48.1
Left	28	51.9
Profession		
Business Owner	6	11.1
Office Job	22	40.7
Manual Labour Worker	16	29.6
IT	5	9.3
Other	5	9.3
Mechanism of Injury		
Sports Injury	11	20.4
Hyperextension	14	25.9
Lifting	24	44.4
Total	54	100.0

The mean day of surgery was at least 10 days from the day of injury. The mean pain VAS score at 6 months after surgery was 0.54±0.50. After 6 months, the mean extension deficit was 2.69° (0-10), the mean flexion was 132° (120-140), the mean supination was 76° (50- 85) and 77° for pronation (80-95). Patients were followed up for 6 months, and the mean MEP Score was 91.43±8.26, which shows excellent functional outcomes in patients (Table 2).

**Table 2:** Functional outcomes of patients after 6 months follow-up

	Mean	S. D.
Extension	2.69	6.348
Flexion	132.87	5.713
Supination	76.30	8.805
Pronation	77.96	4.180
Mayo Elbow Performance Score (MEPS)	91.43	8.264

Complications

We are aware of at least one tendon re-rupture, at least four lateral antebrachial cutaneous nerve paresthesia, one patient had reflex sympathetic dystrophy, superficial wound infection developed in one patient, and no temporary or permanent posterior interosseous nerve palsy.

## Discussion

This study discusses the clinical and functional results of a group of patients who had repaired a ruptured DBT at Heartlands Hospital in Birmingham, UK. The results of the study demonstrated that the mean age of the patients was 51±11.01 years. The dominant side was the right side in the majority of the patients (92.6%) and the surgical side was also right in 48% of the patients. Most of the patients were doing office jobs followed by manual labour. Lifting was the most prevalent cause of injury (44.4%) among patients. These results are in accordance with other studies [7-9].

After 6 months of follow-up, mean extension deficit, flexion, supination and pronation were 2.69°, 132°, 76°, and 77° respectively. These results are consistent with other authors [7-9]. A study reported a mean extension deficit of 2°, flexion 134°, supination 76°, and 77° for pronation, which is comparable to our study [10].

The functional outcome at 6 months was excellent with a mean MEP score of 91.43±8.26 which shows excellent functional outcome in patients and is similar to reported results of other fixation methods [11-13] In another research using MEPS, all 23 patients received a good or exceptional performance with a mean score of 9410. These findings are consistent with other researchers who have reported good to exceptional functional outcomes following surgical repair [14-15].

Our analysis found no evidence of radioulnar synostosis. Our complication rate is 11%, which is comparable to previous studies (10%) [16]. They found no evidence of heterotopic ossification. Another research found an overall complication rate of 19.6%, which is greater than ours. The study revealed eight cases of neuropraxia, two cases of heterotopic ossification, and two cases of re-rupture [9].

This study has limitations, specifically in its design. The study is retrospective in nature, a single-centre-based study and lacks a control group that limits the ability to compare outcomes with alternative treatment techniques. An extended period of follow-up is required to gain a more comprehensive understanding of the long-lasting effectiveness of surgical results and the occurrence of late problems. This can be covered with a prospective, multicentre study with a randomised design. Despite these limitations, this study is both applicable and illustrative of actual practice in smaller institutions with considerable sample size and follow-up with the use of standardized outcome measures.

## Conclusions

Anatomical intramedullary fixation of the DBT results in excellent functional outcomes at 6 months. The anatomical correction led to the recovery of supination strength. This approach allows for the anatomical reinsertion of the distal biceps tendon while reducing the danger of posterior intra-osseous nerve damage.
